# Persistent Sitting and Walking Difficulties After Abdominoperineal Excision and Anterior Resection: Results From the Quality of Life in Rectal Cancer Study

**DOI:** 10.1097/DCR.0000000000003710

**Published:** 2025-03-14

**Authors:** Lina Björklund Sand, Charlotta Larsson, Rode Grönkvist, Eva Haglind, Eva Angenete

**Affiliations:** 1 Department of Surgery, SSORG-Scandinavian Surgical Outcomes Research Group, Institute of Clinical Sciences, Sahlgrenska Academy, University of Gothenburg, Gothenburg, Sweden; 2 Department of Surgery, Region Västra Götaland, Alingsås Lasarett, Alingsås, Sweden; 3 Department of Surgery, Region Västra Götaland, Sahlgrenska University Hospital/Östra, Gothenburg, Sweden; 4 Department of Biostatistics, School of Public Health and Community Medicine, Institute of Medicine, Sahlgrenska Academy, University of Gothenburg, Gothenburg, Sweden

**Keywords:** Abdominoperineal excision, Anterior resection, Perineal symptoms, Rectal cancer, Sitting difficulties, Walking difficulties

## Abstract

**BACKGROUND:**

The main surgical resection options in rectal cancer are anterior resection for tumors in the mid- or upper rectum and abdominoperineal excision for tumors in the lower rectum. A previous study showed long-term persistent perineal symptoms and sitting difficulties after abdominoperineal excision.

**OBJECTIVE:**

To examine the prevalence and extent of sitting and walking difficulties after abdominoperineal excision compared with anterior resection.

**DESIGN:**

An observational, prospective, longitudinal, multicenter, international study.

**SETTINGS:**

Data were collected from participants in the quality of life in rectal cancer study. Participants answered questionnaires about bodily functions, symptoms, and quality of life at baseline and at 1 and 2 years after diagnosis.

**PATIENTS:**

Patients with newly diagnosed rectal cancer, regardless of stage, were included. The study included 1024 patients, of whom 64% underwent anterior resection and 36% underwent abdominoperineal excision.

**MAIN OUTCOME MEASURES:**

The primary objective was to estimate the prevalence and ORs of sitting or walking difficulties between the 2 surgical procedure groups: abdominoperineal excision and anterior resection.

**RESULTS:**

In the group of patients who underwent abdominoperineal excision, 29% had sitting difficulties after 2 years compared with 12% in the group who underwent anterior resection (OR, 2.65; 95% CI, 1.71–4.09; *p* < 0.0001). Walking difficulties after 2 years were reported by 35% after abdominoperineal excision compared with 24% after anterior resection (OR, 1.50; 95% CI, 1.02–2.22; *p* = 0.04).

**LIMITATIONS:**

The observational nature of the study could be regarded as a limitation.

**CONCLUSIONS:**

Abdominoperineal excision was associated with both sitting and walking difficulties among patients with rectal cancer at significantly higher rates compared with anterior resection. It is probable that attention from health care could improve the situation of the patients through enhanced rehabilitation. See **Video Abstract**.

**CLINICAL TRIAL REGISTRATION:**

ClinicalTrials.gov (NCT01477229).

**DIFICULTADES PERSISTENTES PARA SENTARSE Y CAMINAR POSTERIOR A LA ESCISIÓN ABDOMINOPERINEAL Y RESECCIÓN ANTERIOR: RESULTADOS DEL ESTUDIO QoLiRECT:**

**ANTECEDENTES:**

Las principales opciones de resección quirúrgica en el cáncer de recto son la resección anterior para los tumores en el recto medio o superior y la escisión abdominoperineal para los tumores en el recto inferior. Un estudio previo mostró síntomas perineales persistentes a largo plazo y dificultades para la sedestación posterior a la escisión abdominoperineal.

**OBJETIVO:**

Examinar la prevalencia y el grado de dificultades para sentarse y caminar después de la escisión abdominoperineal en comparación con la resección anterior.

**DISEÑO:**

Estudio observacional, prospectivo, longitudinal, multicéntrico e internacional.

**ESTABLECIMIENTOS:**

Se recopilaron datos de los participantes en el estudio QoLiRect. Los participantes respondieron cuestionarios sobre funciones corporales, síntomas y calidad de vida al inicio y tras 1 y 2 años posterior al diagnóstico.

**PACIENTES:**

Se incluyeron pacientes con cáncer de recto de reciente diagnóstico independientemente del estadio. El estudio incluyó a 1024 pacientes, de los cuales el 64 % fueron sometidos a una resección anterior y el 36 % a una escisión abdominoperineal.

**PRINCIPALES MEDIDAS DE RESULTADOS:**

El objetivo principal fue estimar la prevalencia y los odds ratios (OR) de dificultades para sentarse o caminar entre los dos grupos de procedimientos quirúrgicos: escisión abdominoperineal y resección anterior.

**RESULTADOS:**

En el grupo de pacientes sometidos a la escisión abdominoperineal, el 29% tenía dificultades para sentarse tras 2 años posterior a la cirugía en comparación con el 12% en el grupo que fue sometido a resección anterior (OR 2,65, IC del 95% 1,71-4,09, *p* < 0,0001). Las dificultades para caminar posterior a los 2 años se informaron en el 35% después de la escisión abdominoperineal en comparación con el 24% después de la resección anterior (OR 1,50, IC del 95% 1,02-2,22, *p* = 0,04).

**LIMITACIONES:**

La naturaleza observacional del estudio podría considerarse una limitación.

**CONCLUSIONES:**

La escisión abdominoperineal se asoció con dificultades para sentarse y caminar en pacientes con cáncer rectal con tasas significativamente mayores en comparación con la resección anterior. Es probable que la atención médica pueda mejorar la situación de los pacientes mediante una mejor rehabilitación. *(Traducción—Dr Osvaldo Gauto*)

Surgery is the cornerstone of rectal cancer treatment, often in combination with radiotherapy and/or chemotherapy.^1,2^ The most common surgical procedure is total mesorectal excision via anterior resection (AR) for tumors in the mid- or upper rectum or abdominoperineal excision (APE) for tumors in the lower rectum.^3^ APE entails a more extensive surgery and leaves a perineal wound in contrast to AR, which does not leave a perineal wound. An extralevator APE (ELAPE) also involves resection of parts of the levator ani, which creates a larger defect than an APE. ELAPE has been described to improve the oncological outcome by improving the circumferential resection margin and reducing the risk of intraoperative perforation, but it is also associated with an increased risk of perineal wound complications.^4,5^ Reconstruction of the pelvic floor can be performed by primary suture, mesh, or myocutaneous flap. Several studies have found a risk of short-term wound complications resulting in pain and delayed healing after APE.^6–8^ In the long term, persistent perineal morbidity causing pain, sitting disability, and quality of life impacts have been reported.^9–11^ Another long-term complication is the development of a perineal hernia. This may be an underreported condition but has been suggested to be common, especially after ELAPE.^12^

Suggested risk factors for persistent perineal problems include delayed wound healing, radiotherapy, and APE, in particular ELAPE. In a Swedish cohort within the abdominoperineal extralevator resection (APER) study, at least 23% of patients reported continuous perineal symptoms 3 years after APE^9^; however, few studies have compared differences between APE and AR.

The purpose of the present study was to examine the prevalence and extent of chronic perineal and intergluteal symptoms in terms of patient-reported sitting and walking difficulties after APE, compared with AR, within the QoLiRECT (Quality of Life in Rectal Cancer) study, a multicenter, international cohort of patients with colorectal cancer with clinical and patient-reported data.^13^ The primary objective of this secondary study, within QoLiRECT, was to compare the frequency of sitting and walking difficulties, and the secondary objectives were to determine differences in distress and contact with a physiotherapist owing to the experienced discomfort.

## MATERIALS AND METHODS

QoLiRECT is an observational, prospective, longitudinal, multicenter, international study in which patients were included between February 2012 and 2015 at rectal cancer diagnosis before the start of treatment. Sixteen hospitals in Denmark and Sweden participated. Patients answered extensive questionnaires about socioeconomic status, health, lifestyle, bodily functions, cognitive function, and quality of life at baseline and at 1, 2, and 5 years after their diagnosis. Clinical data were collected from the quality registers in Sweden (Swedish ColoRectal Cancer Register) and Denmark (Danish Colorectal Cancer Group). A Strengthening the Reporting of Observational Studies in Epidemiology checklist was used to report the present study.

### Data Collection

Data from baseline and 1 and 2 years were retrieved from the QoLiRECT database.

### Inclusion Criteria

All patients presenting at the participating hospitals with newly diagnosed rectal cancer, regardless of stage at diagnosis and plans for treatment, were eligible for inclusion. In addition, an understanding of the Swedish or Danish language was required to answer the questionnaires.

### Exclusion Criteria

The exclusion criteria were age younger than 18 years at diagnosis and no informed consent received or withdrawal of informed consent.

### Questionnaire

The questionnaire was created for the CoLiRECT study, and the design and validation of this study-specific questionnaire have been described in detail in a previous article.^13^ Briefly, the questionnaire is based on semistructured in-depth interviews with patients with rectal cancer at different stages of the disease. Questions were constructed on the basis of themes identified by content analysis of patient interview transcripts. In addition, appropriate questions from earlier studies were added. The questionnaire was reviewed for its content by an expert panel and was subsequently validated face-to-face with patients. In general, a clinimetric approach was used.^14^ The patients were administered questionnaires by research personnel, and those questionnaies were then answered in writing and returned in prepaid envelopes to the study secretariat.

### Ethical Aspects

The Regional Ethical Review Board in Gothenburg approved the study (Dno. 595-11) as well as the Danish Data Protection Agency (2007-58-0015/HEH.750.89.21). The study was registered with ClinicalTrials.gov (NCT01477229) and the local Data Protection Officer (Register ID 29724).

### Outcome and Adjustment Variables

The primary objective was to compare differences in sitting and walking difficulties after 1 and 2 years between patients who underwent APE and those who underwent AR. EuroQol-5 Dimensions-3 Levels (EQ-5D-3L) questionnaires were used. Sitting and walking difficulties were assessed using the following questions: Have you had trouble sitting in the past month? Have you had trouble walking in the past month? Both questions had 4 response categories: no, not at all; yes, a little; yes, moderately; and yes, a lot. The responses to these questions were dichotomized as “no” or “yes” for the statistical analysis.

Secondary objectives were to compare differences in the frequency and amount of distress arising from sitting or walking difficulties between the 2 procedure groups. These differences were assessed for both sitting and walking with the following question: If you were to have, for the rest of your life, as much difficulty sitting/walking as you did in the past month, how would you perceive it? The question had the following response categories: “not applicable,” “it would not distress me at all,” “it would distress me a little,” “it would distress me moderately,” and “it would distress me greatly.” In addition, distress from sitting difficulties was assessed by asking the following question: If you experienced sitting difficulties during the last month, how often did you have sitting difficulties? The question had the following response categories: “not applicable,” “occasionally when I sat down,” “less than half of the times I sat down,” “more than half of the times I sat down,” and “every time I sat down.” Regarding contact with a physiotherapist, the questionnaire included the following question: Have you, during the last year, been in contact with a physiotherapist? The question has the following response categories: “no,” “yes, I contacted a physiotherapist myself,” and “yes, I was referred to a physiotherapist.”

Primary analyses were adjusted for the following covariates: age, sex, BMI, ASA classification, smoking, risk consumption of alcohol (classified as Alcohol Use Disorders Identification Test-C of more than 5 drinks for men and more than 4 drinks for women), physical activity assessed on the Saltin-Grimby Physical Activity Level Scale, tumor stage, neoadjuvant (chemo)radiotherapy, adjuvant chemotherapy, and minimally invasive surgical procedure. Secondary analyses were unadjusted.

### Statistical Analysis

To examine the specific effect of the type of surgical procedure on sitting or walking difficulties, participants who reported such difficulties at baseline were excluded from the corresponding primary end point analysis. This exclusion gave rise to 2 data sets for analysis: participants with no sitting difficulties at baseline (n = 956) and those with no walking difficulties at baseline (n = 963). A sensitivity analysis for both primary end points using the entire sample was also performed.

Missing values in data were addressed by multiple imputation by chained equations using the “mice” package in R. The models for missing data were specified using both primary end point variables and all adjustment covariates. In addition, missing values in the question regarding walking difficulties were estimated from the responses to the EuroQol-5 Dimensions (EQ-5D) mobility question where possible.

Analyses of the primary end points were performed using logistic regression adjusted for the covariates described earlier. The effects of the primary end point variables were reported as ORs with 95% CIs. Analyses of secondary end points were performed using Brunner-Munzel tests as the shape of response distributions differed between the tested groups. The group differences for secondary end points were reported as the probability of superiority (ie, the probability that a randomly chosen individual who had undergone APE would report greater difficulties than a randomly chosen individual who had undergone AR, with ties broken randomly).

Due to multiple comparisons, the familywise error rate is inflated. However, since the objective of this study was explorative and hypothesis-generating, no correction for multiple testing was performed.

R version 4.3.2 was used to perform statistical analyses and generate graphics. Secondary end point analysis was performed with the “brunnermunzel” package, and graphics were created with the “ggplot2” package.^15^ Directed acyclic graphs identified confounders and were used for adjustment to reduce bias.

## RESULTS

**FIGURE 1.**
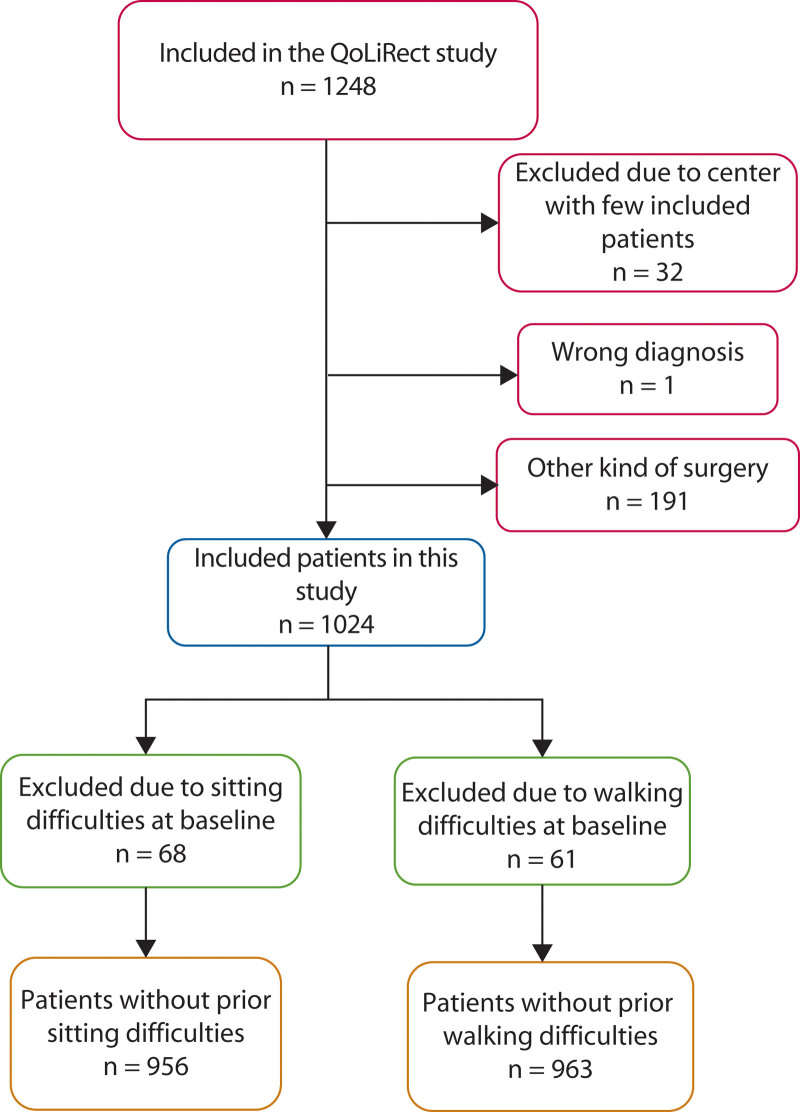
Flow chart showing included and excluded patients in the study.

QoLiRect included 1248 patients between February 2012 and September 2015. For the analyses in this study, 1024 patients were available (Fig. 1). Patients with sitting or walking difficulties at baseline were excluded, which left 956 patients without sitting difficulties and 963 patients without walking difficulties for the primary and secondary end point analyses. The mean age was 67 years, 63% were men, and 36% underwent APE (Table 1). The demographics revealed that patients who underwent APE had more advanced tumors and, hence, neoadjuvant (chemo)radiotherapy was more common in this group. The distribution of surgical resection methods and reconstruction methods in the APE group can be found in Tables 2 and 3. Complications such as infection and anastomotic leakage are available in Table 4. In the AR group, 429 patients (66%) received a temporary loop ileostomy.

**TABLE 1.** Demographics and patient characteristics

*Variable*	*Anterior resection* *(N = 651*)	*Abdominoperineal excision* *(N = 373*)	*Overall* *(N = 1024*)
Sex			
Women	252 (38.7%)	127 (34.0%)	379 (37.0%)
Men	399 (61.3%)	246 (66.0%)	645 (63.0%)
BMI			
Mean (SD)	26 (4.3)	26 (4.2)	26 (4.3)
Missing	66 (10.1%)	43 (11.5%)	109 (10.6%)
Age, y, mean (SD)	67 (10)	68 (10)	67 (10)
Country			
Denmark	168 (25.8%)	78 (20.9%)	246 (24.0%)
Sweden	483 (74.2%)	295 (79.1%)	778 (76.0%)
Tumor cT-stage			
0	0 (0%)	1 (0.3%)	1 (0.1%)
1–2	183 (28.1%)	94 (25.2%)	277 (27.1%)
3	392 (60.2%)	191 (51.2%)	583 (56.9%)
4	49 (7.5%)	77 (20.6%)	126 (12.3%)
Missing	27 (4.1%)	10 (2.7%)	37 (3.6%)
Tumor cN stage			
0	269 (41.3%)	163 (43.7%)	432 (42.2%)
1–2	319 (49.0%)	184 (49.3%)	503 (49.1%)
Missing	63 (9.7%)	26 (7.0%)	89 (8.7%)
Tumor M stage			
0	556 (85.4%)	324 (86.9%)	880 (85.9%)
1	40 (6.1%)	33 (8.8%)	73 (7.1%)
Missing	55 (8.4%)	16 (4.3%)	71 (6.9%)
Comorbidity (ASA class)			
1	164 (25.2%)	82 (22.0%)	246 (24.0%)
2	369 (56.7%)	225 (60.3%)	594 (58.0%)
3	100 (15.4%)	58 (15.5%)	158 (15.4%)
4	5 (0.8%)	2 (0.5%)	7 (0.7%)
Missing	13 (2.0%)	6 (1.6%)	19 (1.9%)
Comorbidity^a^			
0	230 (35.3%)	132 (35.4%)	362 (35.4%)
1	364 (55.9%)	201 (53.9%)	565 (55.2%)
Missing	57 (8.8%)	40 (10.7%)	97 (9.5%)
Physical activity (Saltin-Grimby)			
1	73 (11.2%)	49 (13.1%)	122 (11.9%)
2	393 (60.4%)	228 (61.1%)	621 (60.6%)
3	104 (16.0%)	50 (13.4%)	154 (15.0%)
4	10 (1.5%)	5 (1.3%)	15 (1.5%)
Missing	71 (10.9%)	41 (11.0%)	112 (10.9%)
Smoker			
No	540 (82.9%)	294 (78.8%)	834 (81.4%)
Yes	47 (7.2%)	34 (9.1%)	81 (7.9%)
Missing	64 (9.8%)	45 (12.1%)	109 (10.6%)
Risk consumption of alcohol			
No	558 (85.7%)	303 (81.2%)	861 (84.1%)
Yes	37 (5.7%)	32 (8.6%)	69 (6.7%)
Missing	56 (8.6%)	38 (10.2%)	94 (9.2%)
Neoadjuvant radio(chemo)therapy			
No	328 (50.4%)	77 (20.6%)	405 (39.6%)
Yes	316 (48.5%)	292 (78.3%)	608 (59.4%)
Missing	7 (1.1%)	4 (1.1%)	11 (1.1%)
Adjuvant chemotherapy			
No	268 (41.2%)	229 (61.4%)	497 (48.5%)
Yes	249 (38.2%)	97 (26.0%)	346 (33.8%)
Missing	134 (20.6%)	47 (12.6%)	181 (17.7%)
Minimally invasive surgical technique			
No	335 (51.5%)	183 (49.1%)	518 (50.6%)
Yes	304 (46.7%)	185 (49.6%)	489 (47.8%)
Missing	12 (1.8%)	5 (1.3%)	17 (1.7%)
Surgical procedure			
Anterior resection	551 (84.6%)	0 (0%)	551 (53.8%)
Abdominoperineal excision	0 (0%)	373 (100%)	373 (36.4%)
Hartmann’s operation	100 (15.4%)	0 (0%)	100 (9.8%)
Marital status			
In a relationship	448 (68.8%)	252 (67.6%)	700 (68.4%)
Not in a relationship	145 (22.3%)	82 (22.0%)	227 (22.2%)
Missing	58 (8.9%)	39 (10.5%)	97 (9.5%)
Education			
Lower than high school	192 (29.5%)	115 (30.8%)	307 (30.0%)
High school	171 (26.3%)	98 (26.3%)	269 (26.3%)
Higher than high school	227 (34.9%)	121 (32.4%)	348 (34.0%)
Missing	61 (9.4%)	39 (10.5%)	100 (9.8%)
Trouble sitting at baseline			
No	573 (88.0%)	283 (75.9%)	856 (83.6%)
Yes	20 (3.1%)	48 (12.9%)	68 (6.6%)
Missing	58 (8.9%)	42 (11.3%)	100 (9.8%)
Trouble walking at baseline			
No	555 (85.3%)	307 (82.3%)	862 (84.2%)
Yes	37 (5.7%)	24 (6.4%)	61 (6.0%)
Missing	59 (9.1%)	42 (11.3%)	101 (9.9%)

Data are reported as frequency (percentage) unless otherwise noted.

a Comorbidity was characterized by a number of health conditions, including joint disorders, cardiovascular, neurologic, pulmonary, renal, bowel, and psychological conditions as well as diabetes and chronic pain, and was defined as the presence of at least 1 of these conditions.

ASA = American Society of Anesthesiologists.

**TABLE 2.** Descriptive data regarding the surgical method used in the APE group after 2 y

*APE – surgical method*	*Intersphincteric APE* *(N = 42*)	*Traditional APE* *(N = 101*)	*ELAPE* *(N = 90*)	*Undefined* *(N = 140*)
Sitting difficulties, n/N Missing, n	8/30 12	28/76 25	26/64 26	–
Walking difficulties, n/N Missing, n	14/30 12	26/76 25	23/64 26	–

APE = abdominoperineal excision; ELAPE = extralevator APE.

**TABLE 3.** Descriptive data regarding reconstruction method used in the APE group after 2 y

*Reconstruction method*	*Flap* *(N = 12*)	*Mesh* *(N = 126*)	*Suturing only* *(N = 158*)	*Undefined* *(N = 77*)
Sitting difficulties, n/N Missing, n	2/5 7	36/90 36	32/121 37	–
Walking difficulties, n/N Missing, n	2/5 7	32/90 36	45/123 35	–

APE = abdominoperineal excision.

**TABLE 4.** Surgical complications

*Surgical method*	*Abdominoperineal excision (n = 373*)	*Anterior resection* *(n = 651*)
Any surgical complication,^a^ n (%)	99 (27%)	134 (21%)
Sitting difficulties, n/N Missing, n	23/69 30	18/91 43
Walking difficulties, n/N Missing, n	32/70 29	26/93 41
Wound infection, n (%)	56 (15%)	35 (5%)
Sitting difficulties, n/N Missing, n	15/39 17	6/24 11
Walking difficulties, n/N Missing, n	18/40 16	7/24 11
Intra-abdominal infection, n (%)	23 (6%)	31 (5%)
Sitting difficulties, n/N Missing, n	5/16 7	5/23 8
Walking difficulties, n/N Missing, n	9/16 7	8/24 7
Anastomotic leakage, n (%)	–	43 (7%)
Sitting difficulties, n/N Missing, n	–	8/28 15
Walking difficulties, n/N Missing, n	–	8/29 14

a Registered surgical complication within 30 days after surgery demanding treatment according to Clavien-Dindo classification grade II–V.

### Primary End Points

**FIGURE 2.**
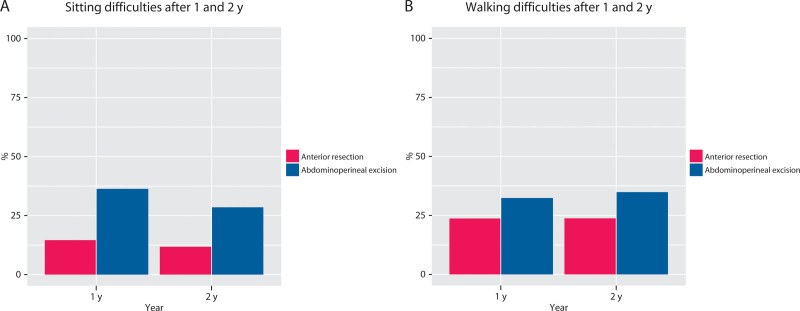
Primary endpoints. A, Sitting difficulties after 1 and 2 y. B, Walking difficulties after 1 and 2 y.

Of patients who had undergone APE, 29% reported sitting difficulties after 2 years, compared with 12% of those who had undergone AR. Walking difficulties after 2 years were reported by 35% of those in the APE group compared with 24% in the AR group (Fig. 2). There was a significant difference between the 2 groups indicating more difficulties after APE, both for sitting (OR, 2.65; 95% CI, 1.71–4.09; *p* < 0.0001) and walking (OR, 1.50; 95% CI, 1.02–2.22; *p* = 0.04) after 2 years (Table 5). The corresponding results after 1 year and after 2 years can be found in Table 5 and Figure 2.

**TABLE 5.** Primary endpoints—summary table

*Endpoint*	*Abdominoperineal* *excision*	*Anterior resection*	*OR*	95% CI	*p*
Sitting difficulties after 1 y	37%	15%	2.99	2.02–4.41	<0.0001
Sitting difficulties after 2 y	29%	12%	2.65	1.71–4.09	<0.0001
Walking difficulties after 1 y	33%	24%	1.42	0.98–2.04	0.06
Walking difficulties after 2 y	35%	24%	1.50	1.02–2.22	0.04

In the APE group, patients who underwent ELAPE with reconstruction with mesh or myocutaneous flap had more sitting difficulties compared to patients who had intersphincteric APE (Tables 2 and 3). Also, patients with wound infections in the APE group seemed to have more sitting difficulties (Table 4).

In the AR group, sitting difficulties were more common if the patients had anastomotic leakage (29% vs 12%, *p* = 0.03).

Among the covariates analyzed, we found that neoadjuvant (chemo)radiotherapy was associated with sitting difficulties after 1 year (OR, 1.97; 95% CI, 1.19–3.27; *p* < 0.01) but not after 2 years (*p* = 0.11). We found no other significant associations regarding sitting difficulties after 1 or 2 years. For walking difficulties, there were associations with age (OR, 1.02; 95% CI, 1.00–1.04; *p* = 0.02), BMI (OR, 1.06; 95% CI, 1.02–1.10; *p* < 0.001), or ASA classification (OR, 1.71; 95% CI, 1.28–2.29; *p* < 0.001) after 1 year. Results were similar after 2 years.

### Secondary End Points

The results regarding frequencies and amount of distress are presented as the probability of superiority for APE over AR (Table 6). Sitting difficulties were significantly greater among patients who had undergone APE compared with AR, and distress was also significantly more pronounced after both 1 and 2 years (Table 6). Distress concerning walking difficulties was also significantly higher after APE.

**TABLE 6.** Secondary endpoints—summary table

*Endpoint*	*Probability of superiority—APE over AR*	*p*
Sitting difficulties often (1 y)	0.62	<0.001
Sitting difficulties often (2 y)	0.59	<0.001
Distress due to sitting difficulties (1 y)	0.6	<0.001
Distress due to sitting difficulties (2 y)	0.58	<0.001
Distress due to walking difficulties (1 y)	0.54	0.019
Distress due to walking difficulties (2 y)	0.55	0.004

APE = abdominoperineal excision; AR = anterior resection.

In the abdominoperineal group, 15% had been in contact with a physiotherapist due to sitting or walking difficulties after 1 year, a proportion similar to that of the AR group (14%).

## DISCUSSION

### Summary

This study found that sitting and walking difficulties were more common among patients with rectal cancer 2 years after APE compared with those who underwent AR. Although several studies have reported pain after APE,^7,10,16^ there are few reports focusing on sitting or walking difficulties, apart from another Swedish national cohort study performed in our research group, where 23% of patients reported sitting difficulties 3 years after APE.^9^ The frequency of sitting difficulties in these 2 studies was similar but the timeframe was somewhat different.

ELAPE appears to impact sitting difficulties with 40% of patients after flap or mesh, whereas patients reconstructed with primary sutures have fewer sitting difficulties, although the subgroups were small. This finding is probably attributed to the difference in the size of the defect, although the granularity in our data makes it difficult to confirm.

Sitting difficulties also seemed to be related to pelvic infection. Sitting difficulties was more common in the APE group than in the AR group, and in the AR group, it was more prevalent after anastomotic leakage. This indicates that it is possible that infectious complications may not only be troublesome in the short-term perspective but may also render long-term disability. Still, not all patients with a perineal wound infection reported sitting difficulties, suggesting that this is probably only part of the explanation for sitting difficulties. Perhaps infection often but not always leads to scar tissue, which could cause sitting difficulties. The greater extent of chronic perineal and intergluteal symptoms reported by patients after APE than those after AR suggests that different rehabilitation programs may be required depending on the type of surgery. Only a minority of patients had contact with a physiotherapist, with no differences seen between the APE and AR groups. It is uncertain what information patients were given regarding postoperative rehabilitation as we did not query them regarding this type of information. The effect of physiotherapy after surgery for rectal cancer may be intuitively seen as beneficial, but its role is still unclear and requires further investigation before being included as a recommendation.

We found that neoadjuvant treatment was associated with sitting difficulties after 1 year but not after 2 years. Older age, high BMI, and high ASA classification were all associated with walking difficulties after both 1 and 2 years, whereas neoadjuvant treatment was not. This is interesting as previous data from the Dutch total mesorectal excision trial indicated that patients who underwent neoadjuvant radiotherapy had more walking difficulties compared with surgery alone.^11^ However, it also showed that walking difficulties are often related to multiple factors. Previous studies have also reported problems with wound complications and delayed healing after APE,^6–9^ and this is often associated with neoadjuvant treatment. Currently, the degree to which neoadjuvant treatment and wound complications contribute to long-term sitting and walking difficulties is uncertain.

### Strengths

The prospective, longitudinal, multicenter design contributes to the overall strength of the study as well as the inclusion of baseline observations. The large, well-characterized cohort with few exclusions provides high-quality data and increases the possibility of generalizing our results. The Swedish ColoRectal Cancer Registry is a validated national registry with 98% coverage.^17^

### Limitations

The observational design of the study could be regarded as a limitation. However, through directed acyclic graphs, possible confounders were identified and used to adjust and reduce bias. Another limitation is the lack of details from registry data, where the details on complications and reconstructive measures are sometimes missing.

### Clinical Importance

The large proportion of patients with rectal cancer affected by persistent sitting and walking difficulties after resection surgery clearly makes this a clinical area that requires further attention to ongoing rehabilitation needs. Resources should be addressed to investigate whether physiotherapy should be a bigger part of the rehabilitation process in the future.

## CONCLUSIONS

Sitting and walking difficulties after APE for rectal cancer were seen at significantly higher rates compared with AR. Attention from health care providers may improve patients’ situations through enhanced rehabilitation. Further interventional trials are underway to examine the importance of physiotherapy to prevent chronic perineal symptoms.

## ACKNOWLEDGMENTS

The authors gratefully acknowledge the excellent work performed by the research nurses at the Scandinavian Surgical Outcomes Research Group and by all those involved in the recruitment of patients at participating hospitals: Sahlgrenska University Hospital Östra; Skaraborg Hospital Skövde; NU Hospital Group, Trollhättan; Central Hospital of Karlstad; Södra Älvsborg Hospital, Borås; Karolinska University Hospital; Örebro University Hospital; Sunderbyn Hospital; Västmanland´s Hospital Västerås; Blekinge Hospital, Karlskrona; Mora Hospital; Helsingborg Hospital; Hvidovre Hospital; Slagelse Hospital; Herlev Hospital; and Roskilde Hospital.
